# Rainfall-conditioned landslide susceptibility mapping in data-scarce regions using an interpretable deep convolutional neural network: a case study of the Jebel Marra volcanic massif, Sudan

**DOI:** 10.1038/s41598-026-52402-0

**Published:** 2026-05-08

**Authors:** Musaab A. A. Mohammed, Abazar M. A. Daoud, Abdelrhim Eltijani, Ali A. Mohieldain, Norbert P. Szabó, Péter Szűcs

**Affiliations:** 1 Faculty of Earth and Environmental Sciences and Engineering, University of Miskolc, Miskolc, 3515 Hungary; 2 College of Petroleum Geology and Minerals, University of Bahri, Khartoum, Sudan; 3 Department of Mineralogy and Geology, University of Debrecen, Egyetem Tér 1, Debrecen, 4032 Hungary; 4 Faculty of Earth Sciences, Red Sea University, Port Sudan, Res Sea State Sudan; 5 Faculty of Petroleum and Minerals, Al-Neelain University, Khartoum, Sudan

**Keywords:** Jebel Marra, Tarsin, Convolutional neural networks, Random forest, Gravity data, Landslide suscibtibility mapping, Risk assessment, Climate sciences, Environmental sciences, Hydrology, Natural hazards

## Abstract

Catastrophic landslides in the Jebel Marra volcanic massif of western Sudan recently caused multiple fatalities and extensive damage in Tarsin village and its surrounding agricultural lands following intense rainfall on 1 September 2025. Although these events pose a recurring threat to vulnerable communities, no previous landslide susceptibility assessment has been conducted in this region. This is largely due to the prolonged armed conflict in Darfur, which has restricted field-based investigations since 2003. In response to these challenges, the current study presents the first regional-scale rainfall-conditioned landslide susceptibility assessment for the Jebel Marra volcanic massif. The analysis integrates multi**-**source geospatial, geological, and geophysical datasets with a deep Convolutional Neural Network (CNN) model. A landslide inventory comprising 350 mapped events was developed using multi-temporal satellite imagery and visual interpretation. Key conditioning factors, including topographic parameters, hydrological characteristics, structural lineament density, vegetation cover derived from the Normalized Difference Vegetation Index (NDVI), and selected anthropogenic indicators, were incorporated into the model. The CNN model, trained and validated using stratified k-fold cross-validation, demonstrated high predictive capability (precision: 0.975, recall: 0.992, area under the curve (AUC): 1) and outperformed a benchmark Random Forest model**.** Feature importance analysis further indicates that elevation, curvature, and lineament density are the most influential conditioning factors controlling landslide occurrence in the study area. The resulting hazard map delineates high and very high hazard zones primarily concentrated in the central volcanic highlands and along major drainage corridors, representing approximately 15–20% of the study area. These findings provide a critical scientific basis for disaster risk reduction, humanitarian planning, and land-use management in this conflict-affected region.

## Introduction

Landslides are among the most pervasive and destructive natural hazards worldwide, claiming thousands of lives annually and causing damage to infrastructure, agricultural systems, and natural ecosystems^1,2^. The occurrence of landslides is governed by a complex interplay of geological structures, geomorphological characteristics, hydrological processes, climatic variables, and anthropogenic disturbances that interact in highly nonlinear ways^3,4^. Small changes in triggering factors can dramatically alter slope stability conditions, leading to sudden catastrophic failures. This inherent complexity makes landslide prediction one of the most challenging problems in natural hazard science^5^. Effective identification and mapping of landslide-prone areas are therefore essential for disaster risk reduction, land-use planning, and the sustainable management of mountainous environments^6^.

Traditionally, landslide suscibitbility mapping has relied on heuristic approaches such as the Analytic Hierarchy Process (AHP), and weighted overlay methods^7–11^, as well as statistical techniques including frequency ratio, weight of evidence, and bivariate statistical analysis that assume predominantly linear or simple parametric relationships between conditioning factors and landslide occurrence^12–17^. While these conventional techniques have contributed to mapping landslide hazard, they are fundamentally limited in capturing the nonlinear interactions, threshold effects, and spatially heterogeneous processes that characterize real-world landslide systems^18^. These limitations have motivated the application of more sophisticated analytical frameworks capable of handling complex, high-dimensional geospatial datasets.

In recent years, machine learning algorithms have transformed landslide hazard mapping by offering flexible, data-driven models that automatically learn complex nonlinear patterns from multisource geospatial data^19–23^. Techniques such as Random Forest, Support Vector Machines, and Gradient Boosting have been extensively employed across diverse settings, demonstrating strong predictive performance^24–27^. However, these machine learning models typically rely on manually engineered features and process each spatial location independently without explicitly accounting for neighborhood context or hierarchical relationships among conditioning factors^28^. The emergence of deep learning architectures, particularly Convolutional Neural Networks, has introduced a paradigm shift by enabling automatic feature extraction directly from raw input data while explicitly preserving spatial structure through hierarchical representation learning^29,30^. Recent applications to landslide hazard mapping have demonstrated superior performance compared to traditional approaches, particularly in complex terrain^31–33^. However, these studies have primarily relied on point-based prediction methods, in which each location is classified independently based on its local attributes. This overlooks the spatial context and relationships with neighboring terrain that often control slope failure processes.

The Jebel Marra volcanic complex in western Sudan’s Darfur region exemplifies a critical research gap in landslide hazard assessment. Rising as the highest peak in the region and recognized as a tentative UNESCO geoheritage site^34^, Jebel Marra is a rugged volcanic massif characterized by steep slopes, deeply incised valleys, and fractured basaltic formations. The combination of this geological complexity with a humid microclimate and intense seasonal rainfall creates conditions highly conducive to slope instability. The vulnerability of local communities was tragically demonstrated in September 2025^35^, when a rainfall-induced landslide struck the village of Tarsin, causing fatalities and severe damage to homes and agricultural fields. Since 2003, the region has been severely affected by ongoing political conflict, which has displaced thousands of residents into the mountainous areas of Jebel Marra and rendered much of the region inaccessible to researchers and humanitarian organizations. This combination of natural susceptibility and human-induced pressures underscores the urgent need for a comprehensive landslide risk assessment in the area.

Previous landslide susceptibility studies have been widely conducted in different parts of the world, particularly in Asia and Europe, where data availability and field access allow the application and validation of both statistical and machine learning approaches. However, such studies remain extremely limited in Africa, and are virtually absent in Sudan, resulting in a significant spatial research gap. Moreover, most existing approaches rely on point-based or pixel-wise classification methods, where each location is treated independently based on its local attributes. This assumption neglects the spatial context and neighborhood relationships that play a critical role in slope instability processes, particularly in complex volcanic terrains. In addition to these methodological limitations, very few studies have incorporated gravity-derived structural information for capturing subsurface geological controls, nor have they applied interpretable deep learning frameworks in data-scarce environments. To address these gaps, this study presents the first high-resolution landslide susceptibility assessment for the Jebel Marra massif using a patch-based convolutional neural network (CNN). The approach integrates multi-source geospatial and geophysical data and captures spatial relationships through neighborhood-based learning. This framework provides a novel solution for landslide hazard mapping in inaccessible and data-limited regions and can serve as a foundational tool for authorities, planners, and humanitarian organizations to prioritize high-risk areas and implement effective risk reduction and resilience strategies.

## Description of the study area

**Fig. 1:**
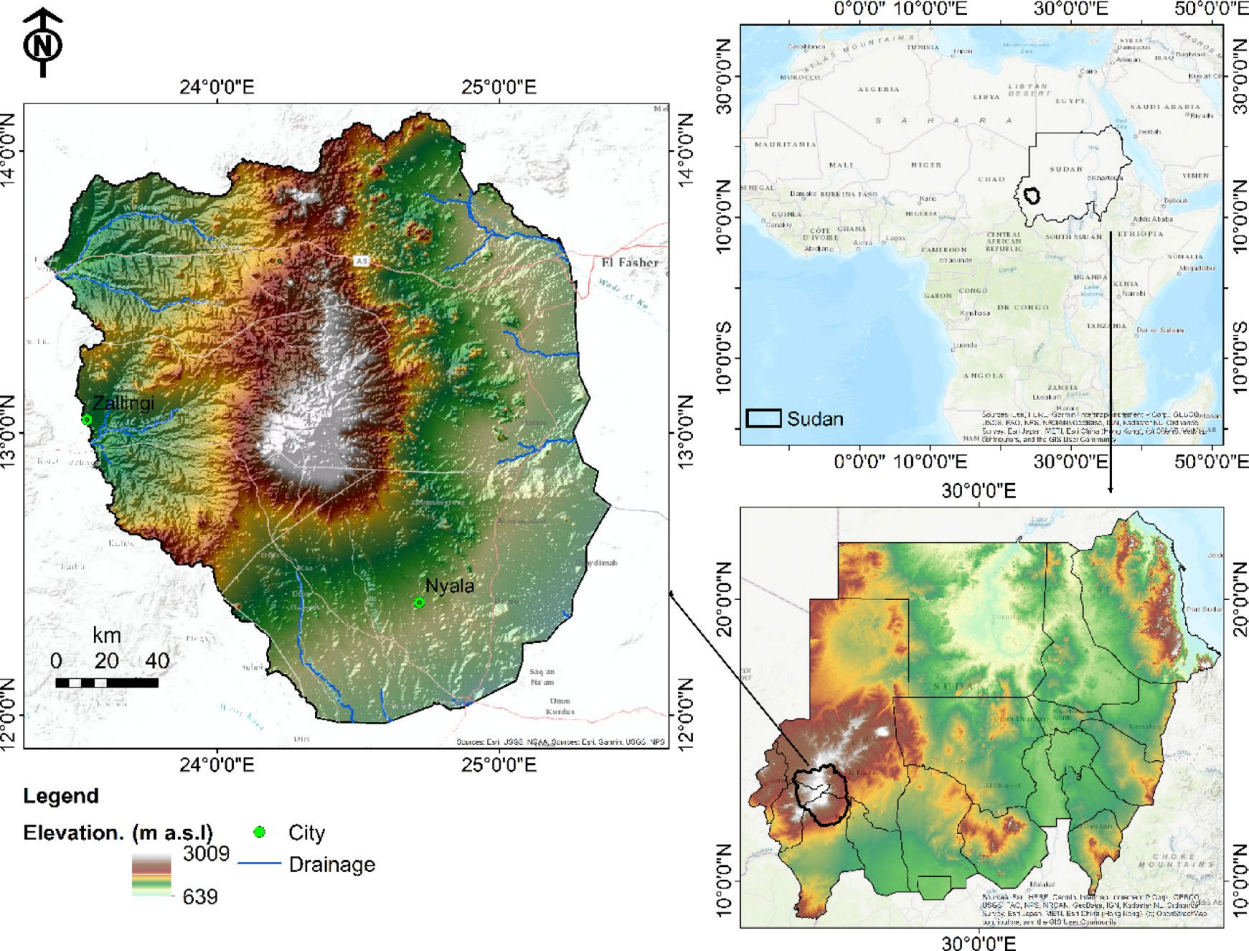
Location map of the study area showing major physiographical features.

The Jebel Marra volcanic massif (12°25′–13°00′ N, 24°10′–24°12′ E) is a prominent geological feature located in the Darfur region of western Sudan, in Northeast Africa (Fig. 1). It rises abruptly from the semi-arid plains of Darfur and forms the country’s highest elevation, reaching approximately 3000 m above sea level. The massif trends northeast–southwest, extends approximately 135 km in length and 80 km in width, and covers an area of about 2000 km^2^. Its physiography includes steep volcanic slopes, radial drainage patterns, and deeply incised valleys, creating terrain inherently predisposed to slope instability.

**Fig. 2:**
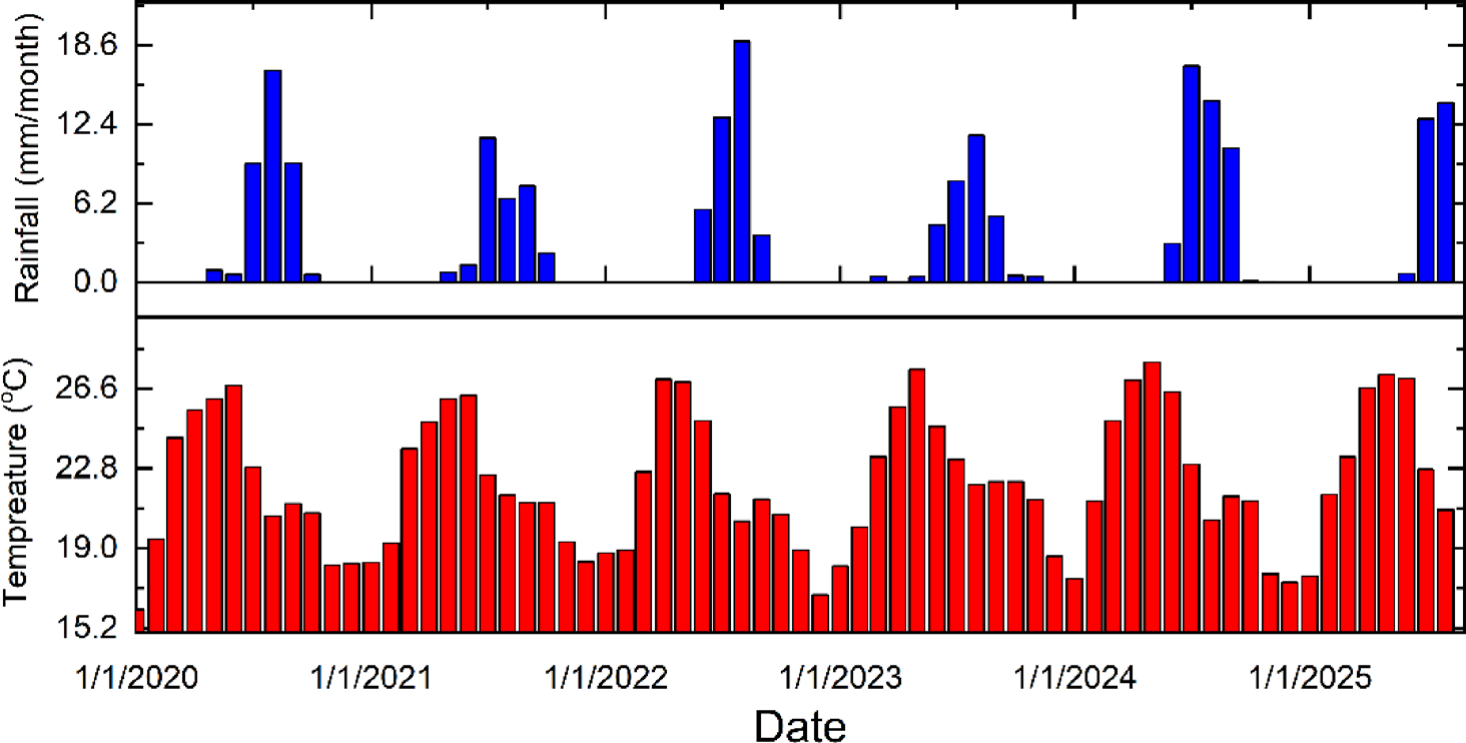
Average monthly rainfall and tempreature between January 2020 to August 2025 obtained from the CHIRPS dataset.

The climatic characteristics of the Jebel Marra massif includeing rainfall and temperature data are illustrated in Fig. 2. A marked seasonal pattern is indicated, with a distinct rainy season extending from June to September, during which the majority of annual rainfall occurs, often in the form of high-intensity rainfall events. Peak rainfall (18 mm/month) is typically recorded in July and August^36^. In contrast, the dry season spans from October to May and is characterized by minimal precipitation and higher temperature variability. Temperature shows moderate seasonal fluctuations between 17 and 27 °C due to the elevated terrain of the massif, with relatively cooler conditions compared to the surrounding lowlands.

Geomorphologically, Jebel Marra is characterized by steep caldera margins, dissected radial valleys, volcanic escarpments, debris-prone slopes, and locally thick weathering mantles developed over fractured volcanic rocks^37^. The rugged topography is further characterized by high relief energy, strong slope gradients, and dense drainage incision, which enhance gravitational stress and facilitate rapid runoff concentration during intense rainfall events. The central and most distinctive geomorphological feature is the Deriba Caldera, a 5-km-wide structure formed by a massive explosive eruption approximately 3500 years ago. Geologicaly, the study area comprise Tertiary volcanic rocks, Quaternary deposits, Nubian Sandstone, and Precambrian basement rocks. The Tertiary volcanic rocks, which dominate the central highlands, consist mainly of fractured basaltic to trachytic lava flows, pyroclastic deposits, and weathered volcanic breccias^38^. These materials exhibit jointed structures, variable porosity, and locally clay-rich alteration zones that reduce shear strength and increase susceptibility to rainfall-induced saturation. Quaternary deposits, consisting mainly of unconsolidated colluvial and alluvial materials, including sandy–silty sediments and clay-rich slope wash deposits^39^. The Nubian Sandstone, widely distributed across Sudan, consists mainly of consolidated sandstones that are susceptible to weathering and erosion. In contrast, the Precambrian basement is composed of crystalline igneous and metamorphic rocks (Gniesses and Gabros)^40,41^. These older basement rocks are locally overlain and intruded by volcanic deposits, creating zones of mechanical weakness along contacts and fractures.The Precambrian basement rocks and Nubian Sandstone formations exhibit greater resistance to failure due to their consolidated lithologies.

## Materials and methods

### Gravity data processing and analysis

**Fig. 3:**
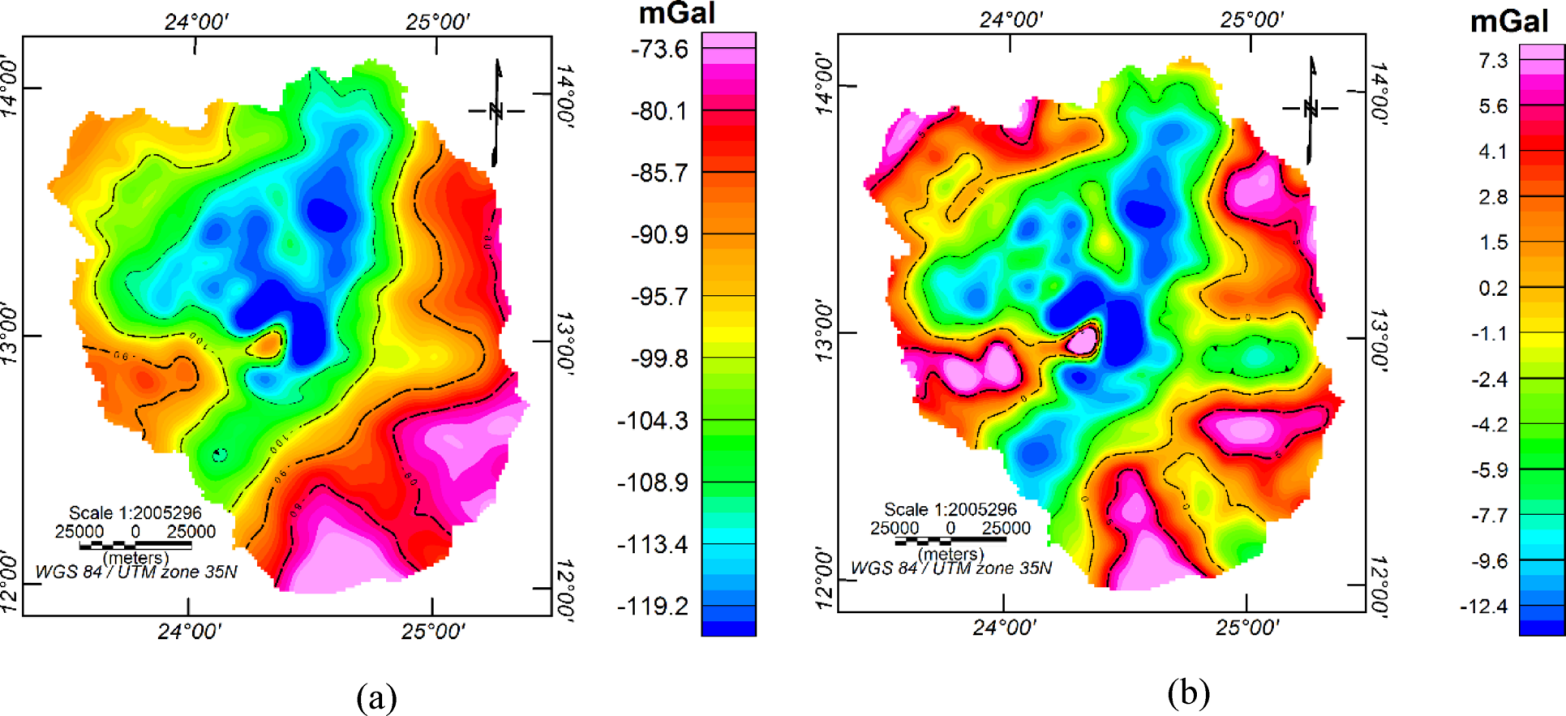
(**a**) Bouguer anomaly map; (**b**) Residual anomalu map.

To investigate the influence of subsurface geological structures on slope stability in the Jebel Marra massif, we utilized satellite-derived gravity data from the EIGEN 6C4 model^42^ to construct a linmeanet map of the study area. This global model incorporates data from the GOCE mission, providing a high-resolution gravity field with a typical standard deviation of 10 mGal for gravity anomalies^43^. The complete Bouguer anomaly (Fig. 3a) represents a composite signal of density contrasts from all subsurface sources. For landslide sucibtibility mapping, isolating the component related to near-surface density variations is critical because these can directly influence slope strength and failure mechanisms. We separated the residual (shallow) gravity signal from the regional (deep) field using upward continuation subtracted from the complete Bouguer anomaly map. The upward continuation process acts as a low-pass filter, attenuating high-frequency signals associated with shallow sources while enhancing longer-wavelength signals from deeper structures^44^. The residual anomaly (Fig. 3b), was then obtained by subtracting this upward-continued regional field from the original Bouguer anomaly (Eq. 1).

In the present study, gravity data were used to delineate subsurface structural discontinuities that act as preparatory controls on landslide occurrence. In fractured volcanic terrains, such structures influence rock-mass integrity, permeability, weathering intensity, and the localization of drainage incision. These effects are particularly relevant under intense rainfall, when structurally weakened slopes are more prone to saturation and failure. Thus, gravity-derived lineaments provide complementary information on tectonic inheritance and subsurface weakening that cannot be obtained reliably from optical imagery alone.

Edge detection techniques are used to delineate geological source boundaries to construct a linmeanet map of the study area. Classical approaches, including the total horizontal derivative and vertical derivative have been widely applied^45,46^. Subsequent advancements include tilt angle filter^47^ and the total horizontal derivative of the tilt angle^48^.

Derivatives of potential field data are utilized as indicators of geological source boundaries; the first vertical derivative (FVD) of the gravity field is effective for identifying shallow structures with low density contrasts^49^. The FVD can be computed either by evaluating the gravity gradient ∂g/∂z directly^50^. The Total Horizontal Derivative (THD) has been extensively applied to identify density contrast boundaries in gravity data^46^. Its main advantage lies in its low sensitivity to noise, as it only requires the computation of the two first-order horizontal derivatives^51^. THD is computed using Eq. 1 as:

1 $$\mathrm{T}\mathrm{H}\mathrm{D}= \sqrt{{\left(\frac{\partial \mathrm{g}}{\partial \mathrm{x}}\right)}^{2}+{\left(\frac{\partial \mathrm{g}}{\partial \mathrm{y}}\right)}^{2}}$$

where ∂g/∂x, ∂g/∂y are the derivatives in x and y, respectively.

The Tilt Angle Derivative (TDR) filter is a robust technique for edge detection in potential field data, including both gravity and magnetic surveys^47^. This filter operates by normalizing the vertical gradient of the potential field with respect to its horizontal gradient, yielding a measure of the tilt or inclination of the field, as expressed in (Eq. 2) as:

2 $$\mathrm{T}\mathrm{D}\mathrm{R}= {\mathrm{tan}}^{-1} \left(\frac{\frac{\partial \mathrm{g}}{\partial \mathrm{z}}}{\sqrt{{\left(\frac{\partial \mathrm{g}}{\partial \mathrm{x}}\right)}^{2}+{\left(\frac{\partial \mathrm{g}}{\partial \mathrm{y}}\right)}^{2}}}\right)$$

The Tilt Angle (TA) filter generates positive values directly above the source and negative values at increasing distances, with the zero-contour reliably highlighting density or susceptibility discontinuities. This property makes the Tilt Angle filter particularly effective for delineating geological boundaries.

The Tilt Angle of the Horizontal Gradient (TAHG) is an edge-detection technique that enhances the interpretation of potential field data^52^. By applying the tilt angle to the horizontal gradient (Eq. 3), TAHG emphasizes source edges through amplitude maxima while balancing contributions from both shallow and deep sources. It is calculated as follows:

3 $$\mathrm{T}\mathrm{A}\mathrm{H}\mathrm{G}= {\mathrm{tan}}^{-1} \left(\frac{\frac{\partial \mathrm{H}\mathrm{G}}{\partial \mathrm{z}}}{\sqrt{{\left(\frac{\partial \mathrm{H}\mathrm{G}}{\partial \mathrm{x}}\right)}^{2}+{\left(\frac{\partial \mathrm{H}\mathrm{G}}{\partial \mathrm{y}}\right)}^{2}}}\right)$$

where $\frac{\partial \mathrm{H}\mathrm{G}}{\partial \mathrm{x}},\frac{\partial \mathrm{H}\mathrm{G}}{\partial \mathrm{y}}, \mathrm{a}\mathrm{n}\mathrm{d} \partial \mathrm{H}\mathrm{G}/\partial \mathrm{z}$ are the two horizontal and vertical gradients of the horizontal gradient, respectively.

The extracted lineaments derived from these gravity-based filters were integrated into the landslide susceptibility model as a proxy for subsurface structural control. These structures represent zones of increased fracturing and reduced mechanical integrity, which enhance slope instability when subjected to intense rainfall.

### Conditioning factors for landslide suscibtiblity mapping

A set of conditioning factors representing topographic, hydrological, geological, environmental, climatic, anthropogenic, and geophysical controls were selected to model landslide in the Jebel Marra region. These factors were derived from multisource datasets to capture both surface and subsurface influences on slope stability (Table 1). Topographic variables including slope, curvature, and elevation, were obtained from the 30 m SRTM DEM, while hydrological parameters, including distance to drainage, were generated through DEM-based hydrological analysis. Geological factors such as lithology and lineament density were compiled from the national geological map and gravity-derived structural interpretation. Lineament density represents fracture concentration, whereas distance to major lineaments captures the spatial influence of tectonic discontinuities on slope stability. Environmental indicators, including land cover and NDVI, were extracted from the ESRI 2024 Land Use/Land Cover (LULC) dataset available through ArcGIS Living Atlas, with a spatial resolution of 10 m. This annually updated dataset is derived primarily from Sentinel-2 imagery and was selected to represent the most recent land-surface conditions in the study area. Climatic variables (rainfall and temperature) were obtained from the CHIRPS dataset (annual mean 2020–2025), which were used to characterize recent spatial rainfall variability across the study area rather than long-term temporal trends. Anthropogenic influence was represented by distance to roads from OpenStreetMap. All factors were resampled to a uniform 30 m spatial resolution and projected to the WGS 84 coordinate system (EPSG:4326) to ensure spatial consistency for CNN processing (Table 1).

**Table 1:** Description of the datasets used in the present study.

Category	Factor	Description / Source
Topographic	Slope, Curvature, Elevation	Derived from SRTM DEM (30 m)
Hydrological	Distance to drainage	Extracted from DEM hydrological analysis
Geological	Lithology, Lineament density	From the national geological map and gravity-derived lineaments
Environmental	Landcover, NDVI	From ESRI LULC imagery (2024)
Climatic	Rainfall	From CHIRPS dataset (annual mean 2020–2025)
Anthropogenic	Distance to roads	From OpenStreetMap

### Inventory map generation

**Fig. 4:**
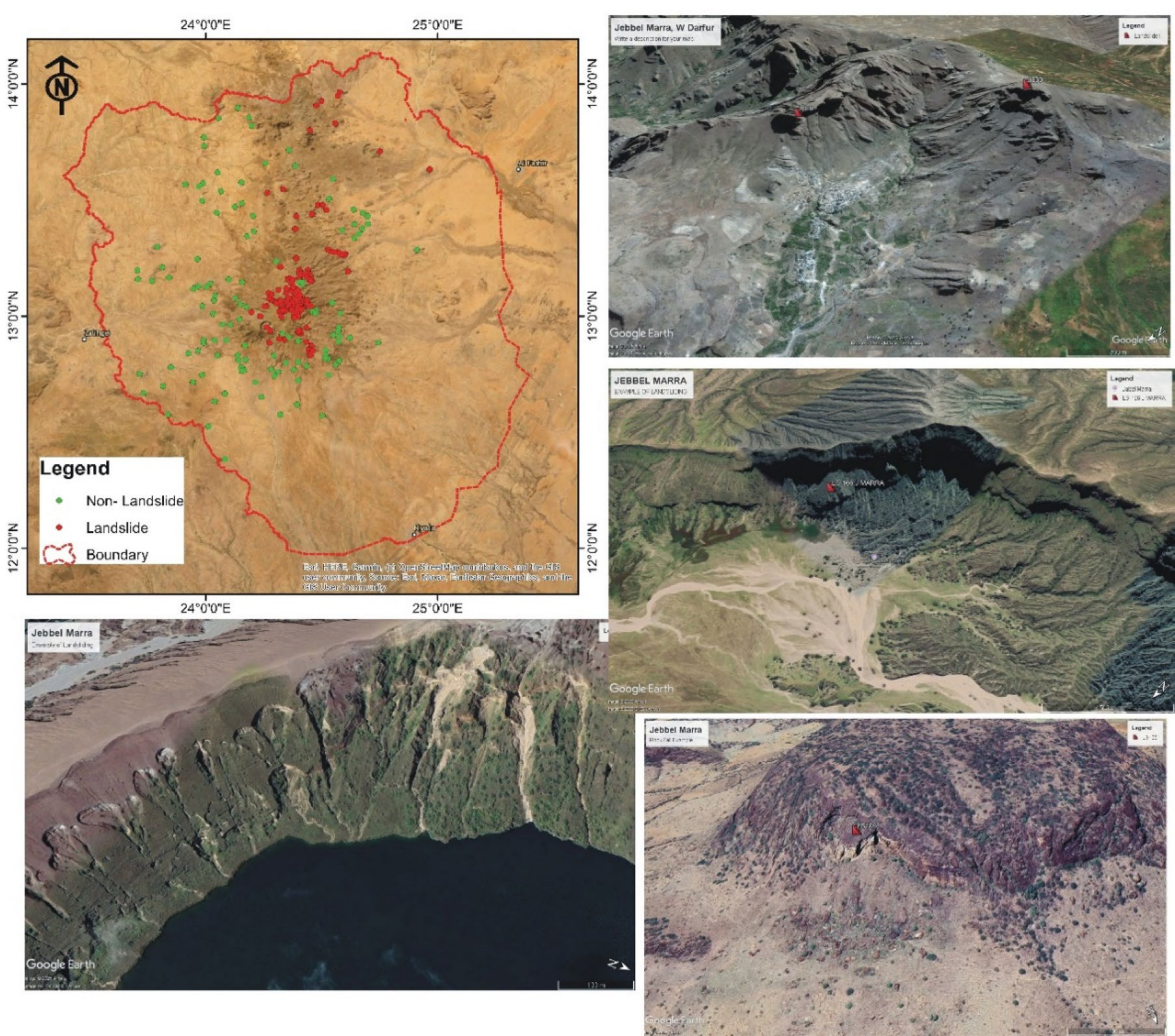
Inventory map of Jebel Marra showing exact areas of landslides using Google Earth Pro.

**Fig. 5:**
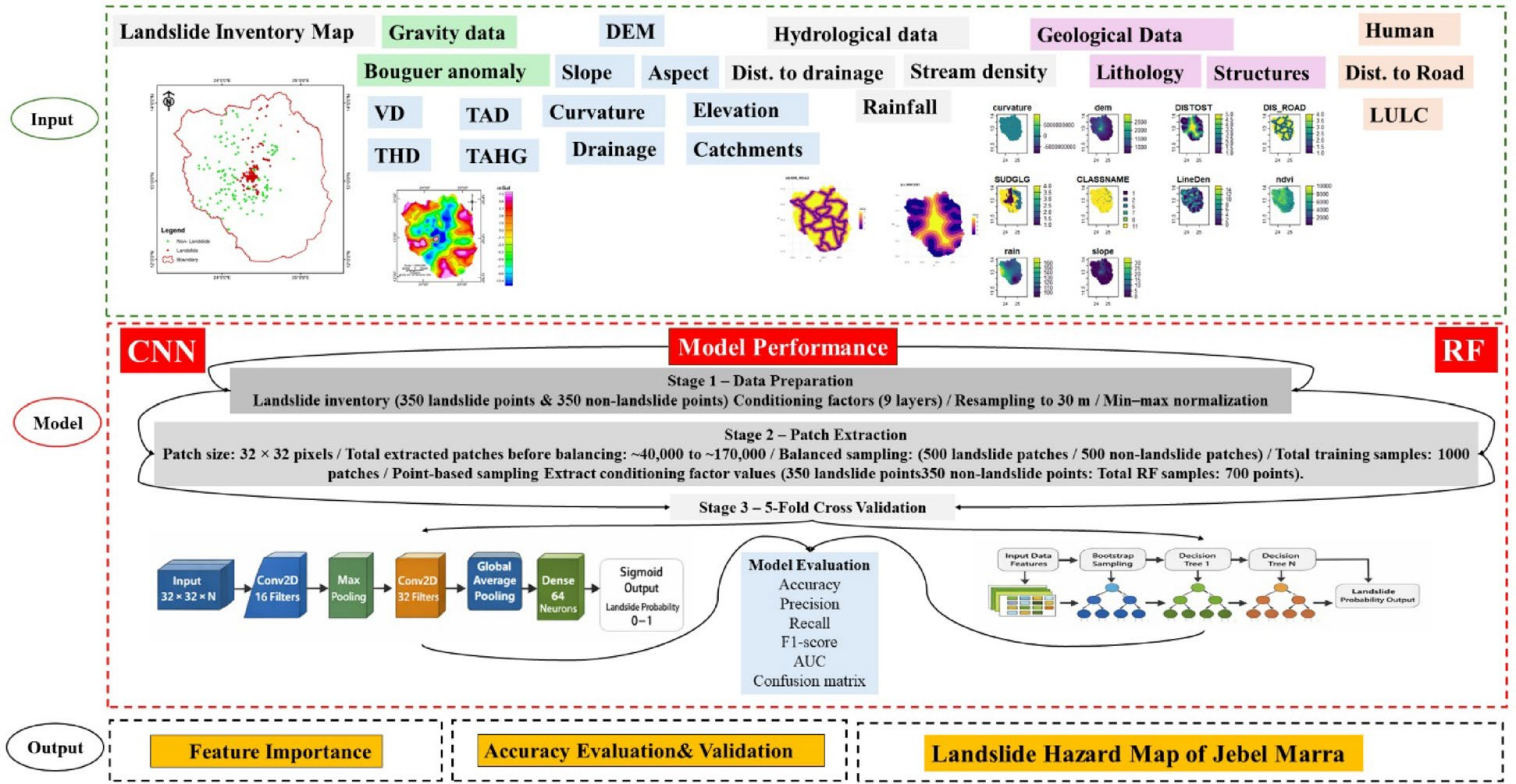
The Flowchart illustrates the data and methods applied in the present study.

Because of ongoing conflict in the study region, field access was severely limited. To develop a reliable landslide inventory, we employed high-resolution imagery from Google Earth Pro to visually identify landslide occurrences as well as stable (non-landslide) locations (Fig. 4). The most recent landslide event (Tarsin, 1 September 2025) served as a reference to guide identification of characteristic slope failures, scarps, and disrupted terrain. Using Google Earth Pro imagery, landslide points were digitized by examining shadows, exposed soil/debris, slope contrast, linear scarps, and other geomorphological signatures visible from imagery (Fig. 5). Non-landslide (absent) points were selected from similar terrain, lithology, elevation, and slope, but showing no evidence of mass movement in the imagery. This remote inventory approach has precedence in literature. For example,^53^ used Google Earth to build polygon-based landslide inventories in the Bandung Basin, Indonesia, by visual interpretation of high-resolution satellite imagery, similarly,^54^ detailed landslide geomorphology mapping in Qinghai, China, using Google Earth imagery.^12^ combined Google Earth imagery with field surveys for identifying landslide sites in the Chemoga watershed in Ethiopia .

The inventory thus produced for Jebel Marra includes 350 landslide points and balanced number of non-landslide points. All landslide and non-landslide locations were digitized from Google Earth Pro, which uses the WGS 84 geographic coordinate system (EPSG:4326). Accordingly, the landslide inventory was initially referenced in UTM WGS 84 Zone 35N. Uncertainties were acknowledged in areas with lower image resolution or cloud cover, but the Tarsin event provided a recent, high-quality benchmark for mapping

### Convolutional neural network (CNN)

Convolutional Neural Networks (CNNs) are deep learning models that identify spatial patterns and hierarchical features in image data^55^. These models are effective for tasks requiring both local detail and broader spatial context, making them suitable for geospatial applications. In this study, a CNN was used to predict the probability of landslide occurrence based on geo-environmental conditioning factors. The CNN architecture consists of several specialized layers for feature learning. Convolutional layers extract spatial features such as edges, textures, and patterns relevant to landslide-prone areas (Fig. 5). Pooling layers reduce the dimensionality of feature maps, retaining essential information and lowering computational cost. A global average pooling layer aggregates spatial features into a compact representation, allowing the model to focus on feature presence rather than position. Fully connected layers integrate the learned features and produce the probability of landslide occurrence.

The Convolutional Neural Network (CNN) was selected as the primary model for landslide susceptibility mapping due to its capacity to automatically learn complex spatial patterns and nonlinear relationships from multi-factor raster data^32^. Unlike traditional statistical models, which often assume linear or predefined relationships between conditioning factors, CNNs can adaptively extract relevant features directly from input maps. This makes them particularly suitable for areas like the Jebel Marra Volcanic Massif, characterized by rugged topography, heterogeneous geology, and limited landslide inventory data.

### Random forest (RF)

Random Forest (RF) is an ensemble machine learning algorithm that constructs multiple decision trees and combines their outputs to enhance prediction accuracy and robustness^56^. Each tree is trained on a randomly selected subset of the data and predictor variables, which reduces overfitting and improves generalization. The final prediction aggregates the results of all trees, using majority voting for classification or averaging for regression. RF is effective for high-dimensional datasets, captures complex non-linear relationships, and provides measures of variable importance, making it widely used in geospatial studies^24,25,57^. In this study, RF was selected as the benchmark model because it is among the most widely used machine-learning approaches in landslide susceptibility studies, performs well with nonlinear relationships and mixed predictors, and provides a reliable non-spatial baseline against which the added value of CNN-based spatial feature learning can be evaluated.

### Model development and evaluation

The development of the rainfall-conditioned landslide susceptibility model involved several stages including data preparation, preprocessing, model training, validation, and generation of the final susceptibility map. The analysis began with the preparation of the input datasets, which consisted of a landslide inventory and multiple environmental conditioning factors. The landslide inventory was converted into a binary raster dataset where pixels with a value of 1 represented known landslide locations and pixels with a value of 0 represented stable terrain. The conditioning factors were compiled into a multi-band raster stack representing the spatial predictors used for model training.

A simplified CNN architecture was designed to extract spatial patterns associated with landslide occurrence from multi-layer conditioning-factor patches. The network consists of two convolutional layers with 16 and 32 filters, respectively, each followed by batch normalization and max-pooling operations to reduce spatial dimensionality and improve feature stability. A global average pooling layer aggregates the learned feature maps before passing them to a fully connected dense layer with 64 neurons. To reduce overfitting, L2 regularization and dropout were incorporated into the network. The final output layer uses a sigmoid activation function to estimate the probability of landslide occurrence. The model was trained using the Adam optimizer and binary cross-entropy loss function, which are commonly used for binary classification problems.

To maintain spatial consistency across all datasets, the rasters were aligned to a common reference grid defined by the conditioning factor stack. The landslide inventory and study area boundary mask were resampled to match the projection, spatial resolution, and spatial extent of the factor layers. Each conditioning factor was subsequently normalized using min–max scaling to transform the values into a range between zero and one. Because landslide occurrences represent a minority class compared with stable terrain, the dataset exhibited a strong class imbalance. To reduce model bias toward the dominant non-landslide class, a balanced sampling strategy was implemented. Equal numbers of positive (landslide) and negative (non-landslide) samples were randomly selected from the study area, with a maximum of 1270 samples per class. This balanced sampling approach improves classification stability and increases the model’s sensitivity to landslide-prone areas^58^.

To evaluate the predictive capability of the proposed model, a five-fold cross-validation strategy was implemented^59^. The dataset of extracted image patches was randomly divided into five subsets of approximately equal size. During each iteration, four subsets were used for training and one subset was reserved for validation. This procedure was repeated five times so that each subset served once as the validation set. Such cross-validation helps ensure that the model performance is robust and not dependent on a single data partition. Importantly, validation samples were completely excluded from the training process in each fold, preventing data leakage^60^. Data augmentation techniques, including horizontal and vertical flipping, were applied only to the training samples to improve model generalization while maintaining the independence of validation data.

The final susceptibility map was generated by applying the trained CNN model to every valid pixel within the study area. For each pixel, the normalized conditioning factor values were extracted and provided as input to the model, which produced a probability score ranging from 0 (very low hazard) to 1 (very high susceptibility). These probability values were subsequently mapped to generate the spatial distribution of landslide susceptibility across the study area. The resulting continuous susceptibility map was classified into five discrete hazard classes (very low, low, moderate, high, and very high) using the natural breaks (Jenks) method.

To improve model interpretability, feature importance was analyzed using a gradient-based attribution method. Specifically, the gradients of the model loss with respect to the input feature channels were computed using TensorFlow’s automatic differentiation framework. The mean absolute gradient values were then calculated across spatial dimensions and samples to estimate the relative contribution of each conditioning factor to the model predictions. This approach highlights which input variables most strongly influence the CNN’s decision-making process, allowing a better understanding of the environmental controls on landslide occurrence.

Model performance was assessed using several evaluation metrics derived from the confusion matrix. The Area Under the Receiver Operating Characteristic Curve (AUC) was calculated to measure the model’s ability to distinguish between landslide and non-landslide conditions across different classification thresholds. Additional metrics including accuracy, precision, recall, and F1-score were also computed to provide a comprehensive assessment of classification performance. These metrics collectively evaluate both the predictive accuracy of the model and its ability to correctly identify landslide occurrences while minimizing false detections.

4 $$\mathrm{A}\mathrm{c}\mathrm{c}\mathrm{u}\mathrm{r}\mathrm{a}\mathrm{c}\mathrm{y}=\frac{\mathrm{T}\mathrm{P}+\mathrm{T}\mathrm{N}}{\sum \mathrm{T}\mathrm{P}+\mathrm{F}\mathrm{P}+\mathrm{T}\mathrm{N}+\mathrm{F}\mathrm{N}}$$

5 $$\mathrm{P}\mathrm{r}\mathrm{e}\mathrm{c}\mathrm{i}\mathrm{s}\mathrm{i}\mathrm{o}\mathrm{n}=\frac{\mathrm{T}\mathrm{P}}{\mathrm{T}\mathrm{P}+\mathrm{F}\mathrm{P}}$$

6 $$\mathrm{R}\mathrm{e}\mathrm{c}\mathrm{a}\mathrm{l}\mathrm{l}=\frac{\mathrm{T}\mathrm{P}}{\mathrm{T}\mathrm{P}+\mathrm{F}\mathrm{N}}$$

7 $$\mathrm{F}1\;\mathrm{S}\mathrm{c}\mathrm{o}\mathrm{r}\mathrm{e}=\frac{2 \times \mathrm{P}\mathrm{r}\mathrm{e}\mathrm{c}\mathrm{i}\mathrm{s}\mathrm{i}\mathrm{o}\mathrm{n}\times \mathrm{R}\mathrm{e}\mathrm{c}\mathrm{a}\mathrm{l}\mathrm{l}}{\mathrm{P}\mathrm{r}\mathrm{e}\mathrm{c}\mathrm{i}\mathrm{s}\mathrm{i}\mathrm{o}\mathrm{n}+\mathrm{R}\mathrm{e}\mathrm{c}\mathrm{a}\mathrm{l}\mathrm{l}}$$

where TP is the correctly predicted landslide samples, TN is the correctly predicted non-landslide, FP are the non-landslide misclassified as landslides, and FN is the landslide misclassified as non-landslides.

## Results

### Structural mapping from gravity data

**Fig. 6:**
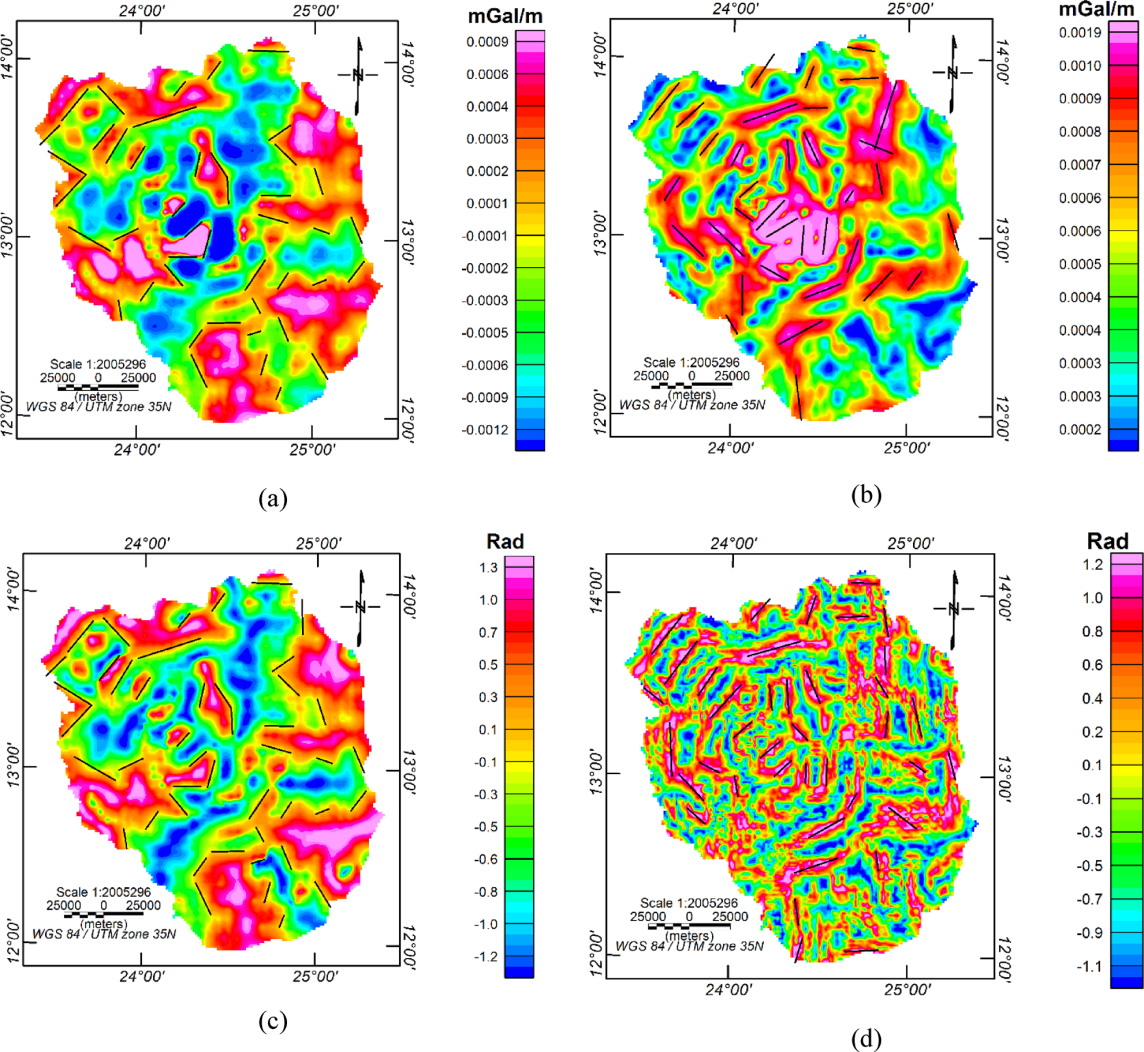
Derivative maps derived from gravity data of the study area: (**a**) First Vertical Derivative (FVD); (**b**) Total Horizontal Derivative (THD); (**c**) Tilt Angle Derivative (TDR); and (**d**) Tilt Angle of the Horizontal Gradient (TAHG). These maps highlight subsurface structural features and delineate lineament trends and lithological boundaries.

Gravity data were processed using multiple edge detection techniques to delineate subsurface structures controlling slope stability in the Jebel Marra region. The applied methods included the first vertical derivative (FVD), total horizontal derivative (THD), tilt angle derivative (TDR), and the horizontal gradient of tilt angle derivative (TAHG) (Fig. 6a–d). The FVD (Fig. 6a) enhanced short-wavelength anomalies, revealing closely spaced, high-frequency anomalies interpreted as near-surface lineaments. In contrast, the THD (Fig. 6b) enhanced the lateral extent of gravity gradients, effectively outlining fault boundaries and lithological contacts. The TDR (Fig. 6c) normalized the effects of shallow and deep anomalies, enabling consistent detection of lineaments across depths, while the TAHG (Fig. 6d) sharpened structural trends, resolving zones of complex intersection. All delineated lineaments were compiled into a comprehensive structural map. Analysis indicates a dominant set of NW–SE and NE–SW trending structures, consistent with the Pan-African orogenic history and subsequent reactivations. Localized E–W lineaments likely reflect younger volcanic-tectonic activity associated with the Deriba caldera formation. The superposition of these trends confirms multiple deformation episodes that continue to influence landscape evolution.

### Analysis of landslide conditioning factors

**Fig. 7:**
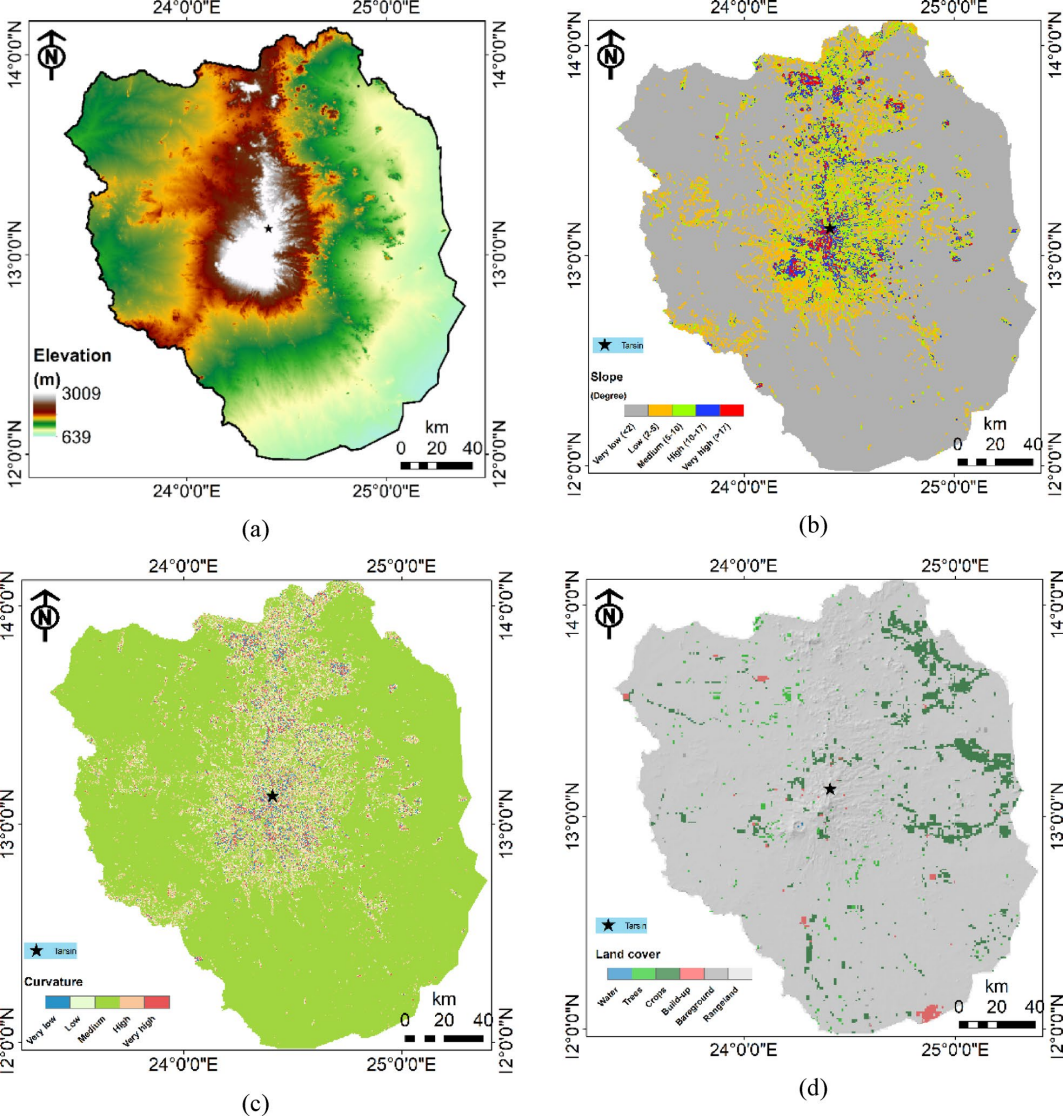
Spatial distribution maps of key topographic and land surface characteristics of the study area: (**a**) Elevation (m); (**b**) Slope (degree); (**c**) Curvature; and (**d**) Land Use/Land Cover (LULC) classification. The maps illustrate the geomorphological variation and land cover heterogeneity across the region.

Topographical factors were extracted and analyzed to assess their influence on landslide initiation (Fig. 7a–d). Elevation (Fig. 7a) ranges from approximately 639 to over 3000 m above sea level across the study area. Higher elevations in the central volcanic massif, particularly around the Deriba caldera and its flanks, coincide with areas of deeply weathered volcanic rocks. These conditions make the slopes more prone to instability^61^. Conversely, lower elevations in the surrounding plains are relatively stable but can still be affected by mass wasting processes due to localized slope undercutting. Slope (Fig. 7b) is a critical determinant of slope stability. Gentle slopes (0–3°) dominate the plains, whereas moderate slopes (3–10°) act as transitional zones that may accumulate colluvium and become unstable under prolonged rainfall. The steepest slopes (> 10°), particularly those concentrated in the central highlands and along caldera rims, exhibit the highest hazard. These steep gradients promote downslope movement by reducing shear strength and increasing the gravitational driving forces^62^.

Curvature (Fig. 7c) provides additional insight into slope geometry and water concentration. Convex slopes, typically associated with ridge tops, tend to shed water and are less susceptible to saturation-induced failure. Concave slopes, on the other hand, concentrate surface runoff and subsurface flow, thereby increasing pore water pressure and the likelihood of failure^63^. The curvature map shows numerous concave features within the highland region, many of which coincide with mapped landslide occurrences. Landcover (Fig. 7d) further modulates slope stability through the role of vegetation and anthropogenic activities. Vegetated areas, particularly those dominated by trees, contribute to slope reinforcement by root cohesion and interception of rainfall. In contrast, bare ground and built-up areas are highly susceptible due to the lack of protective vegetation cover and increased runoff from impervious surfaces. The landcover map of Jebel Marra reveals that the area is dominated by rangeland where most landslides are concentrated.

**Fig. 8:**
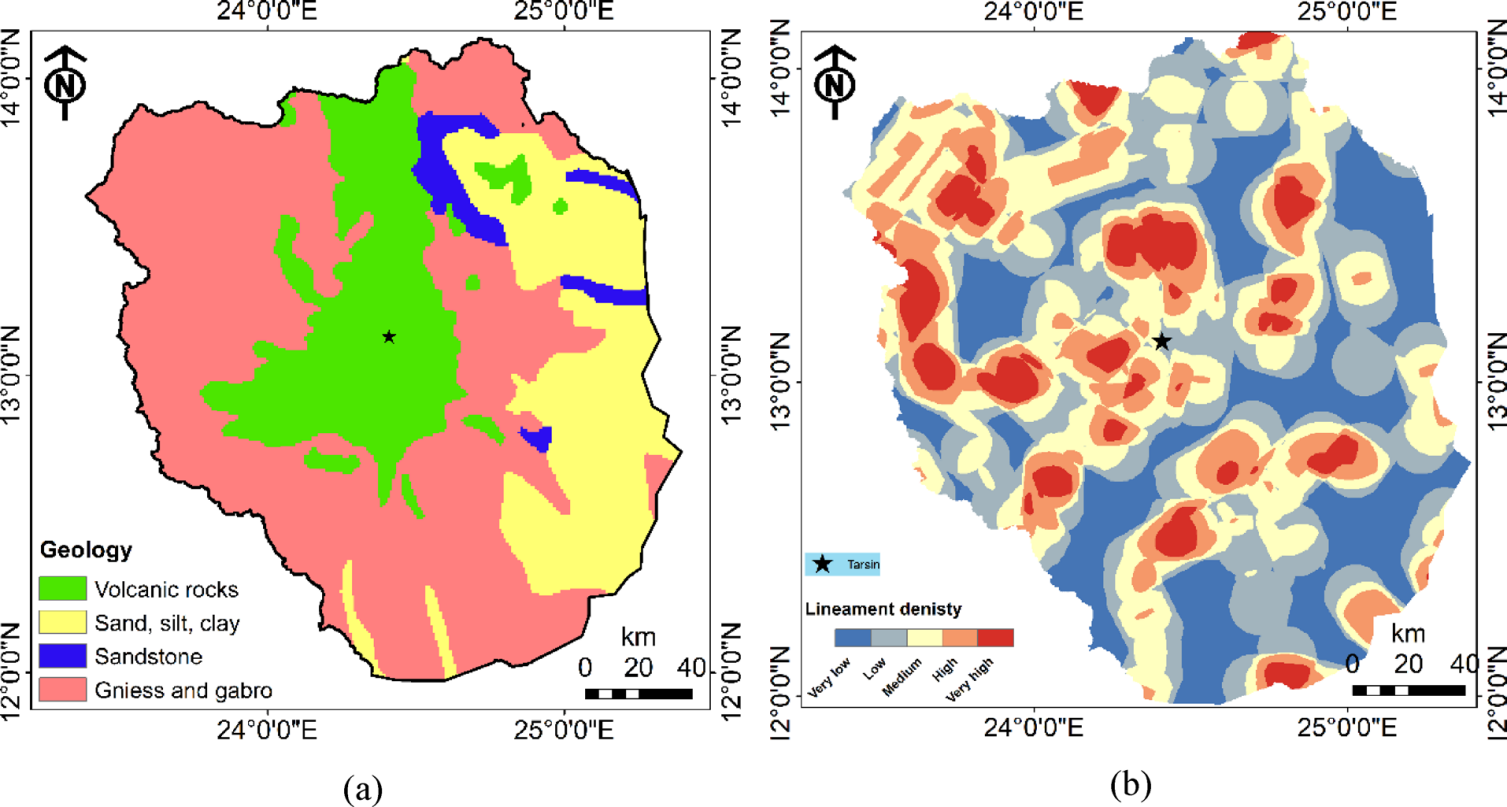
Geological and structural characteristics of the study area: (**a**) Geological map showing the distribution of major lithological units; and (**b**) Lineament density map classified into low, medium, and high density zones.

Geological and structural settings are fundamental in controlling slope stability, as lithological composition and tectonic features largely determine the mechanical behavior of slopes under external stresses^64^. In this study, two primary geological conditioning factors, including geology and lineament density, were incorporated into the landslide susceptibility assessment (Fig. 8a and b).

The study area encompasses diverse geological units, including Tertiary volcanic rocks, Quaternary deposits (Sand, silt clay), Nubian Sandstone, and Precambrian basement rocks (Gniess and gabro) (Fig. 8a). In the hazard assessment, geological units were characterized based on their lithological composition and slope-stability-relevant material properties. The Tertiary volcanic rocks, which dominate the central highlands, consist mainly of fractured basaltic to trachytic lava flows, pyroclastic deposits, and weathered volcanic breccias. These materials exhibit jointed structures, variable porosity, and locally clay-rich alteration zones that reduce shear strength and increase susceptibility to rainfall-induced saturation. Quaternary deposits, consisting mainly of unconsolidated colluvial and alluvial materials, including sandy–silty sediments and clay-rich slope wash deposits, also exhibit high hazard due to their weak cohesion and rapid strength loss when saturated. In contrast, the more competent Precambrian basement rocks and Nubian Sandstone formations exhibit greater resistance to failure due to their consolidated lithologies. However, localized instabilities may occur along weathered zones or faulted contacts. The lineament density map of Jebel Marra (Fig. 8b) shows clusters of low and high-density zones. Lineament density is widely recognized as an important proxy for structural discontinuities such as faults, fractures, and zones of weakness. In volcanic and tectonically active terrains like the Jebel Marra massif, these features significantly influence slope stability by increasing rock mass permeability, facilitating water infiltration, and reducing shear strength along discontinuities^65^. As a result, areas with higher lineament density are more susceptible to landslide initiation.

**Fig. 9:**
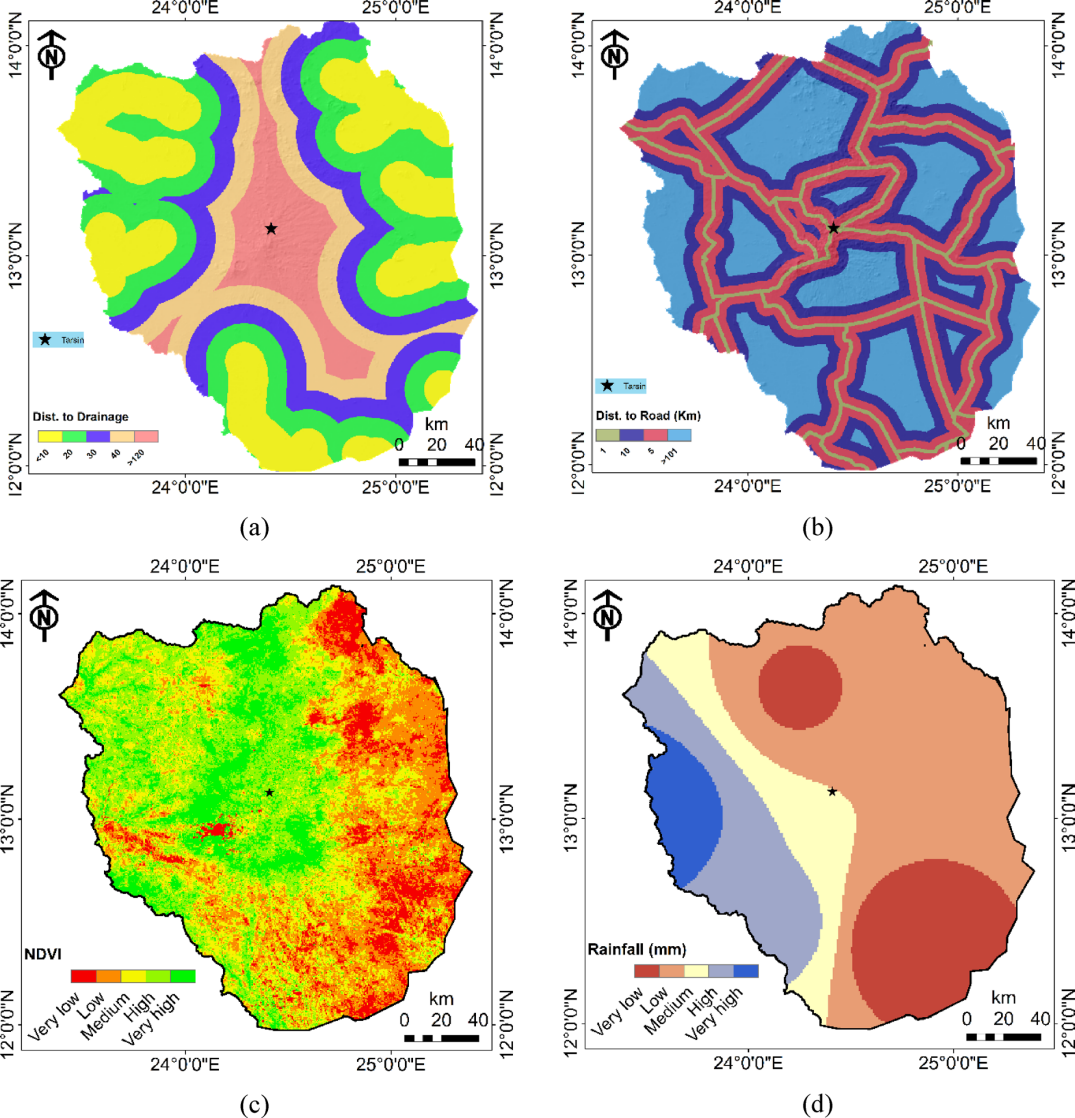
Spatial distribution maps of key environmental variables within the study area: (**a**) Distance to drainage networks (in km); (**b**) Distance to road infrastructure (in km); (**c**) Normalized Difference Vegetation Index (NDVI); and (**d**) Annual rainfall distribution (in mm). All maps are georeferenced using WGS 84/UTM Zone 35N coordinate system (EPSG:32635).

The environmental and anthropogenic conditioning factors that influence landslide hazard in the Jebel Marra volcanic massif include distance to drainage, distance to roads, NDVI, and rainfall (Fig. 9). The distance to the drainage network reveals a radial pattern emanating from the central highlands, with values ranging from less than 10 km to more than 120 km (Fig. 9a). Areas within 10–20 km of major streams tends to be less prone to landslide occurrence, as rainfall is more effectively drained, reducing surface water accumulation and lowering pore-water pressure on slopes. In contrast, the absence or poor development of drainage channels increases the likelihood of mudslides, as rainwater collects on slopes, infiltrates the soil, and triggers shallow failures under saturated conditions. The road network distribution shows a complex pattern with most areas falling within 5–10 km of road infrastructure, while more remote areas exceed 10 km distance (Fig. 9b). Roads significantly influence landslide hazard in multiple ways. Road construction activities involving cutting and filling operations alter natural slope geometries and create over steepened slopes. Areas in close proximity to roads (less than 5 km) would demonstrate elevated landslide hazard, particularly along road cuts in mountainous terrain. However, the relationship may be complex, as roads also provide access for monitoring and mitigation efforts.

The NDVI map displays predominantly high vegetation cover across the massif, with values ranging from approximately − 0.03 to 1 (Fig. 9c). The uniformly high NDVI across most of the region suggests healthy vegetation cover, which generally provides a stabilizing influence on slopes. Vegetation reduces landslide hazard through root systems that mechanically bind soil particles and increase shear strength, canopy interception that reduces the amount of rainfall reaching the ground surface, evapotranspiration that removes moisture from the soil profile, and surface roughness that slows runoff velocity and promotes infiltration. However, areas with lower NDVI values would be more vulnerable to landslides due to reduced root reinforcement and increased surface erosion. On the other hand, the distribution of the average annual rainfall between 2020 and 2025 (Fig. 9d) reveals a consistent spatial gradient, with the lowest precipitation in the southern portion of the massif and the highest rainfall in the western sector. Higher rainfall areas experience increased soil saturation that reduces effective stress and shear strength, elevated pore water pressures that decrease slope stability, greater erosive force from surface runoff, and enhanced weathering of bedrock and soil materials.

### Landslide susceptibility map modeling and validation

**Fig. 10:**
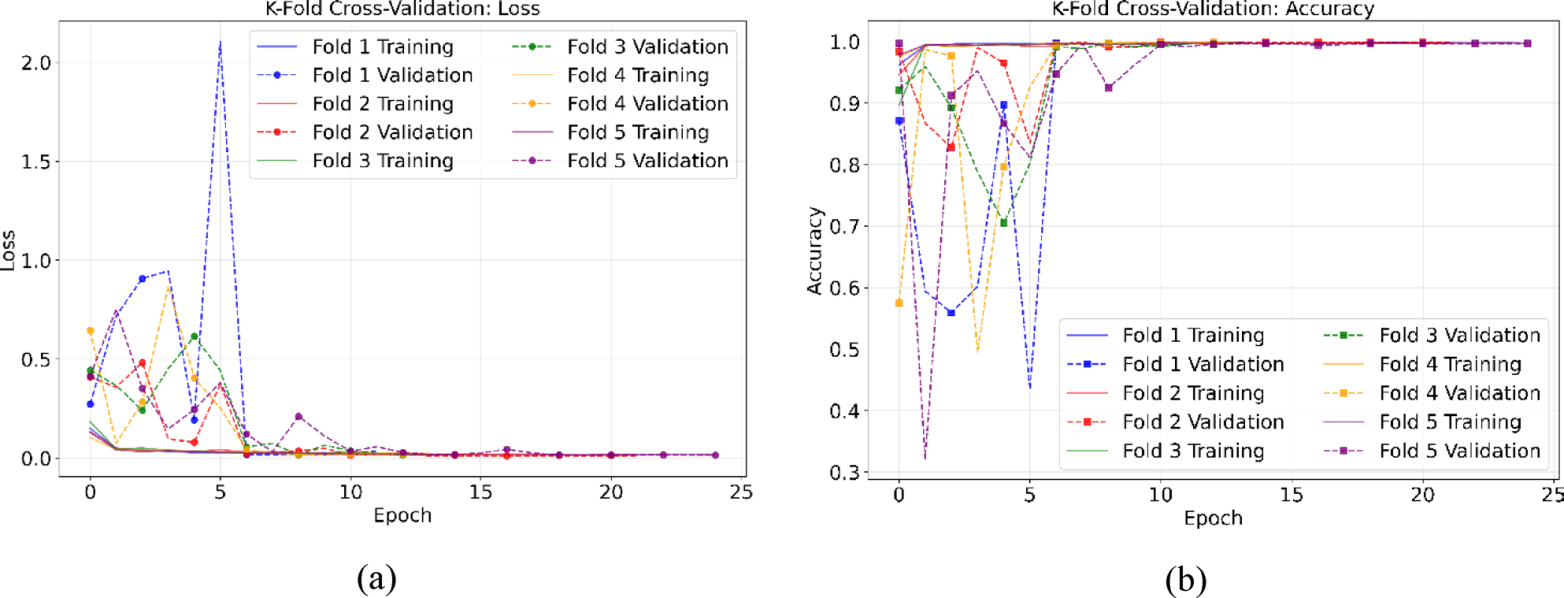
(**a**) K-fold cross validation of loss and (**b**) Accuracy.

**Fig. 11:**
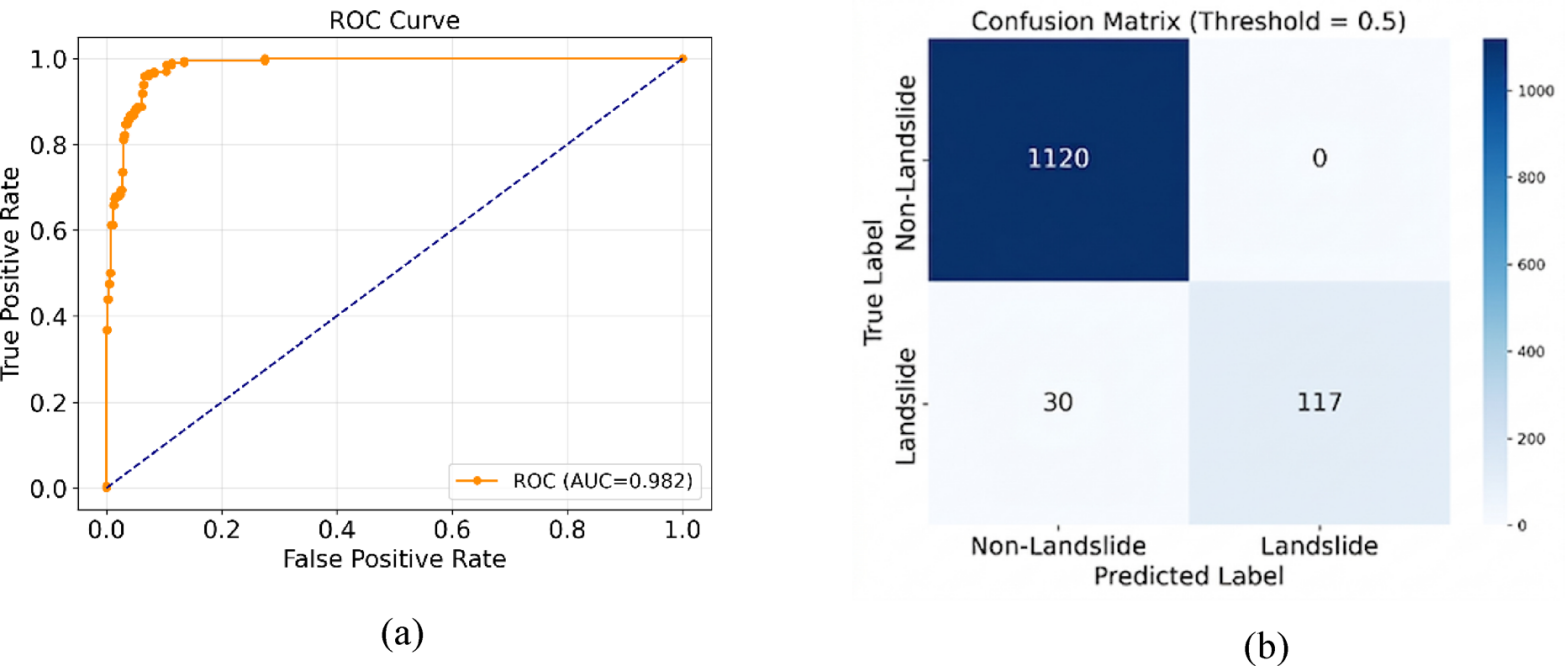
(**a**) The final ROC curve and (**b**) confusion matrix for the model during the validation stage.

The CNN model was developed for the first time in Sudan to assess landslide susceptibility mapping in the Jebel Marra region using conditioning factors derived from topographic, geological, and anthropogenic factors. To ensure robustness and generalization, the model was trained and validated using a k-fold cross-validation strategy. In this procedure, the dataset was partitioned into 5 equal subsets, and the model was iteratively trained on 4 folds while tested on the remaining fold. This rotation reduced the risk of overfitting and bias associated with a single data split. Across all folds, the CNN demonstrated stable convergence of the binary cross-entropy loss function (Figs. 10a and 11). The training and validation losses decreased steadily during the initial epoch before stabilizing near zero, with minimal variability across folds (Fig. 11). This smooth convergence indicates that the model effectively captured spatial and geomorphological patterns without overfitting to specific folds. The low mean validation loss, coupled with the small standard deviation, confirms the stability and generalization capability of the CNN across different data partitions.

**Fig. 12:**
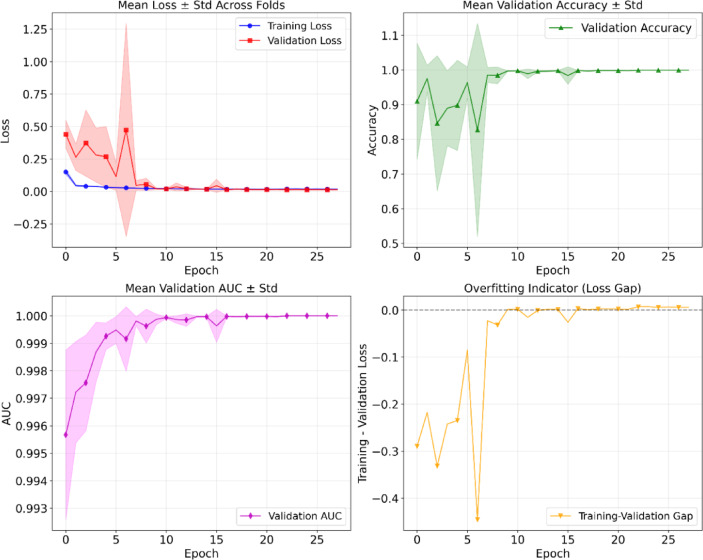
Mean loss across folds, mean accuracy validation, standard deviation of AUC, and overfitting indicator.

Validation accuracy remained consistently high across epochs, reaching near-perfect values after approximately 10 epochs and maintaining this stability thereafter (Figs. 10b, 11 and 12). The ROC curve indicates an excellent predictive performance of the landslide susceptibility model, with an AUC value of 0.982, showing a strong ability to discriminate between landslide and non‑landslide areas across different thresholds. This high AUC reflects a high true positive rate achieved with a very low false positive rate. The confusion matrix at a threshold of 0.5 further confirms this strong performance, correctly classifying 1120 non‑landslide pixels and 117 landslide pixels, with only 30 non-landslide misclassified and no landslide falsely predicted as non-landslides. The mean validation accuracy across folds exceeded 0.982, with very minor fluctuations. The mean validation AUC curve further highlights the exceptional discriminative ability of the CNN. AUC values rapidly increased in the early epochs and plateaued close to 1.0, with negligible variance across folds. The overfitting indicator (training–validation loss gap) remained close to zero after the initial epochs (see Fig. 12), confirming that the CNN generalized well to unseen data.

When trained on the complete dataset, the final CNN model achieved good predictive performance. Evaluation of the confusion matrix revealed that the model correctly classified all non-landslide samples, with no false positives, while only three landslide points out of a total of 122 were misclassified as non-landslides. This level of performance was reflected by a precision of 0.975, indicating a very low false-positive rate; a recall of 0.992, demonstrating the model’s effectiveness in capturing nearly all landslide occurrences; and an F1-score of 0.983, representing an excellent balance between precision and recall. Furthermore, RF model was implemented as a baseline to benchmark the performance of the proposed CNN-based susceptibility mapping. The RF algorithm achieved an overall accuracy of 91.0%, precision of 0.895, recall of 0.962, F1-score of 0.927, and an AUC of 0.964.

**Fig. 13:**
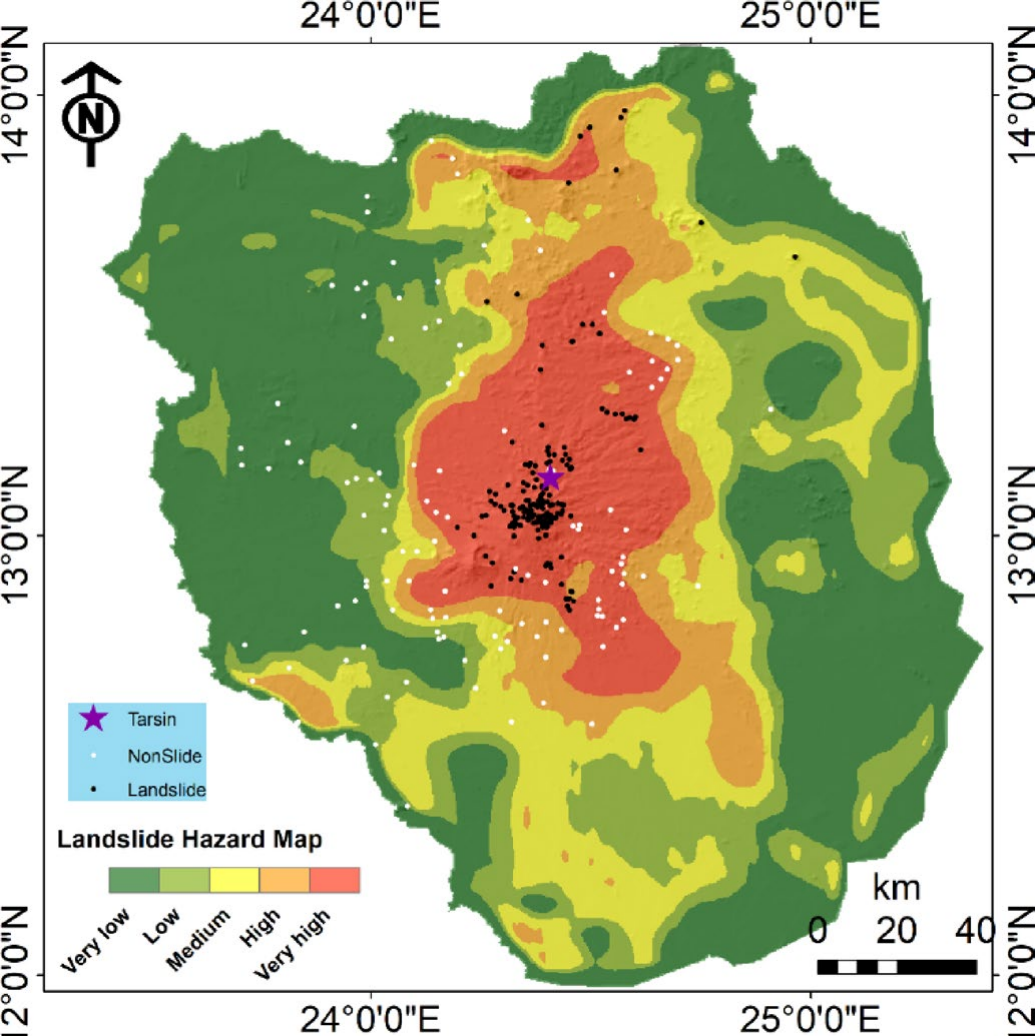
Landslide susceptibility map of Jebel Marra generated by CNN and classified using natural breaks method.

The final landslide susceptibility map for Jebel Marra reveals a distinctive spatial pattern characterized by a pronounced concentration of high hazard zones in the central and upper portions of the volcanic massif, creating a core of elevated risk surrounded by progressively lower hazard zones toward the periphery (Fig. 13). The high-hazard zones are predominantly concentrated across the central and northeastern highlands, forming elongated, fault-aligned corridors that follow the primary drainage networks and structurally weakened slopes. These zones, covering approximately 15–20% of the total area, coincide with regions of steep gradients, deep valleys, and intense dissection, where the interaction between slope steepness and rock mass fracturing provides optimal conditions for slope failure. Several settlements and villages lie within or adjacent to these hazardous corridors, including Tarsin (northeast Jebel Marra, where a destructive landslide already occurred on 1 September 2025), Tonga (southeast), Iro (north), Konga (northwest), Srung (north), Suni (northeast), Sortoni (north), Koinga, Eima, and Tongoro. The concentration of these communities within high-risk belts highlights the growing vulnerability of local populations to slope instabilities, particularly under intensifying rainfall and ongoing land-use pressures. Areas of moderate hazard form transitional belts surrounding the high-risk cores and extending along secondary drainage systems and moderate slope gradients, occupying nearly 30–35% of the total mapped area (Topo, Maya, Tbra, Durgei, Munu, Nertiti, Tonga, Grlnbang). In contrast, low-hazard zones dominate the peripheral plains and lower elevations, encompassing roughly half of the region, where gentle slopes and more coherent lithological units contribute to slope stability.

## Discussion

The present study generated the first landslide susceptibility map for the Jebel Marra volcanic massif using a CNN trained on a comprehensive dataset from remote sensing and geophysical analysis. The input parameters for the CNN model were selected based on their established influence on landslide processes and the availability of reliable spatial data. While these factors capture the primary controls on landslide susceptibility, other potentially influential variables such as soil depth, root cohesion, and human activities were not included due to insufficient spatial data. However, the CNN model demonstrated strong predictive performance, achieving an AUC of 1.00 and an F1-score of 0.983. The high AUC values reflect the strong discriminative capability of the CNN model when combining multiple conditioning factors with spatial context. Similar high predictive performances have also been reported in recent deep-learning-based landslide susceptibility studies using patch-based CNN frameworks. The CNN outperformed the benchmark RF model, consistent with recent studies showing that advanced, spatially aware deep learning architectures have surpassed traditional machine learning algorithms^66,67^. For example, a hybrid XGBoost-RF model has demonstrated high accuracy (AUC > 0.95) in regional-scale assessments, which is lower than the performance observed in the present study by^68^ and^69^. The capacity of CNNs to automatically learn hierarchical spatial features enabled them to capture the complex, non-linear relationships characteristic of the Jebel Marra volcanic landscape. Consistent performance across all k-fold cross-validation folds, with stable convergence and minimal overfitting, further supports the robustness and generalizability of the model^2,70^.

Most historical landslide points are situated within high-hazard corridors, providing substantial evidence for the model’s accuracy. The Tarsin landslide of September 2025, which occurred within a high-hazard zone, serves as a critical validation point and demonstrates the model’s practical relevance for identifying areas of acute hazard. This retrospective prediction enhances confidence in the map’s predictive capability for previously unaffected but highly susceptible areas. However, several important discrepancies between the predicted hazard zones and the observed landslide inventory are evident. Some non-landslide points appear within high-hazard zones, and conversely, some documented landslide points occur in areas classified as low hazard^71^. These inconsistencies must be understood within the context of the inventory development methodology. The landslide inventory for Jebel Marra was compiled entirely from remote-sensing interpretation of satellite imagery, due to the complete absence of ground-based field verification^53^. The Darfur region, where Jebel Marra is located, has experienced prolonged political conflict and security instability since 2003, rendering extensive areas virtually inaccessible to researchers and field teams.

**Fig. 14:**
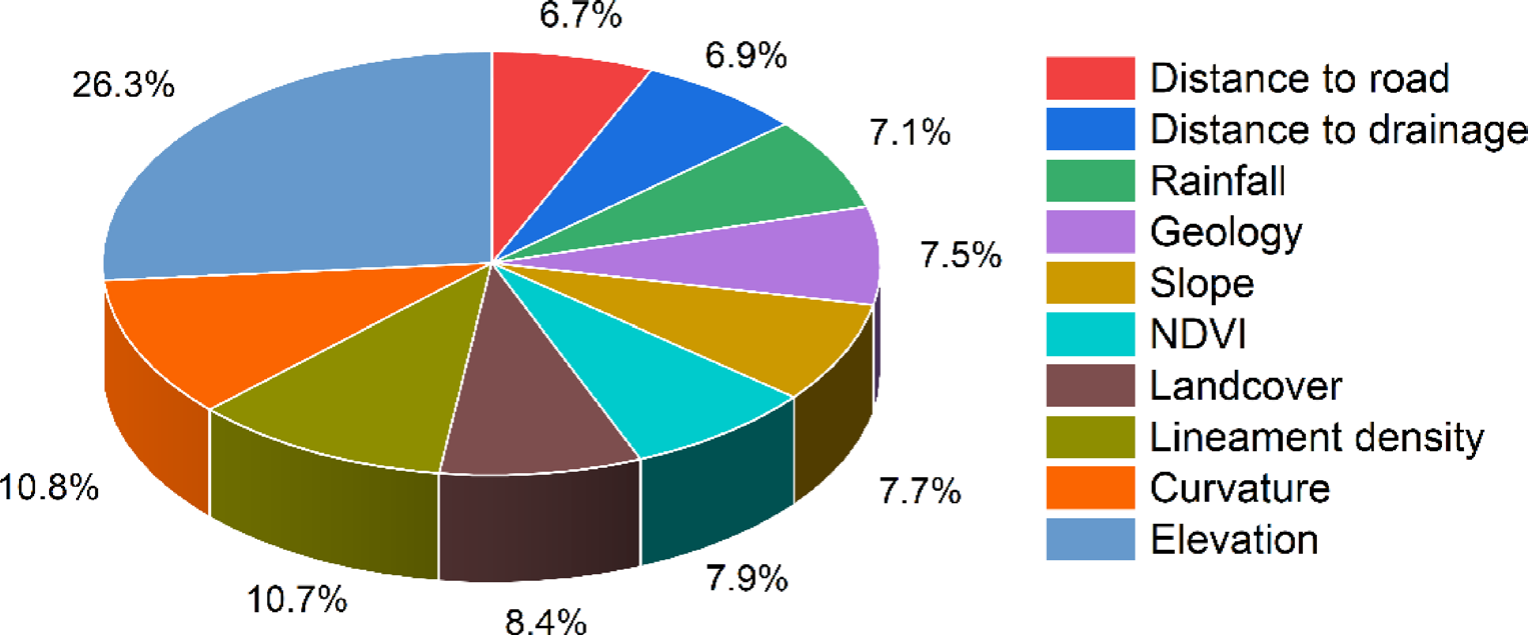
Feature importance of individual conditioning factors of CNNs.

Feature importance analysis identified elevation (26.3%), Curvature (10.8%), and lineament density (8.4%) as the primary factors contributing to the landslides (Fig. 14). Topographic and hydrological factors are dominant in controlling landslide hazard mapping. Elevation is the most influential factor, highlighting the importance of regional-scale topography, where higher elevations are associated with steeper slopes and deeper weathering profiles. Curvature significantly influences local slope geometry and subsurface water concentration, making concave features reliable predictors of failure. The interaction between large-scale elevation and local-scale curvature is evident in the map, as high-susceptibility zones closely align with the steep, incised valleys of the central highlands. Subsurface structures are also critical determinants of landslide susceptibility. The high weighting of Lineament Density confirms that tectonic inheritance is a fundamental control as indicated by^72^.

The moderate feature importance of rainfall (7.1%) may initially seem counterintuitive and warrants detailed interpretation. While rainfall is widely acknowledged as a primary landslide trigger^73^, its relatively low contribution in the spatial susceptibility map reflects the CNN modeling context. The model is designed to identify where landslides are likely to occur based on the spatial variation of conditioning factors. Rainfall data for Jebel Marra, although important, show relatively low spatial variability across the massif’s highland core, where landslides are concentrated. The rainfall gradient, while significant for defining ecological zones, is not pronounced enough over the steep, high-elevation terrain to serve as the primary differentiator for failure location. The entire central highland area receives rainfall at or above a critical threshold sufficient to trigger failures. Thus, while the absolute amount of rainfall serves as the dynamic trigger, its spatial distribution lacks strong discriminatory power to identify the precise locations of failure within this uniformly susceptible zone. The model, therefore, assigns greater importance to factors with higher spatial contrast, such as elevation and curvature, which more effectively identify specific mechanically weak areas within the rain-affected region.

The findings have important implications for landslide risk management in the Jebel Marra massif. Most high-elevation terrain is hydrologically connected and highly susceptible to failure during major rainfall events. This suggests that early warning systems and disaster preparedness strategies should consider rainfall thresholds across the entire massif, rather than targeting only specific sub-areas. The identification of high-risk zones can also guide land-use planning, agricultural management, and infrastructure development to minimize exposure in the most vulnerable areas.

Despite the strong predictive performance of the proposed CNN model, several limitations should be acknowledged. The first and most important limitation relates to field accessibility. The Jebel Marra region has remained severely restricted since 2003 because of the prolonged armed conflict in Darfur, making systematic field verification of landslide locations, failure types, lithological conditions, and slope-material properties impossible during the present study. Consequently, the landslide inventory had to be compiled entirely from remote sensing interpretation of Google Earth Pro imagery and other satellite-based sources. Although this approach is justified in data-scarce and conflict-affected regions, it inevitably introduces uncertainty related to positional accuracy, visual interpretation, and inventory completeness. Small landslides, vegetation-obscured failures, shallow translational movements, and older degraded scars may not have been detected, while some erosional or anthropogenic landforms may have been mistakenly interpreted as landslides.

A second group of limitations is related to the input datasets. The rainfall dataset used in this study represents a relatively short recent period and was incorporated mainly to capture spatial precipitation variability and event-scale triggering thresholds. In addition, some potentially important variables, including detailed geotechnical parameters, soil thickness, groundwater conditions, and discontinuity orientation, could not be included because reliable spatial datasets were unavailable. Finally, the validation framework remains constrained by the available remotely derived inventory and the absence of independently field-verified event datasets. Future research should prioritize field-based validation when security conditions permit, together with higher-resolution DEMs, UAV mapping, InSAR deformation monitoring, longer rainfall time series, and more detailed lithological and geotechnical data. Incorporation of Frequency–Area Distribution (FAD) analysis will enable evaluation of the completeness of the landslide inventory, helping to identify potential underrepresentation of smaller events and reduce biases associated with incomplete landslide datasets. will provide a more comprehensive evaluation of landslide risk, particularly in terms of estimating the probability of large, high-impact events..

## Conclusions

This study provides the first detailed landslide susceptibility assessment for the Jebel Marra volcanic massif in western Sudan, a region recently affected by catastrophic landslides that caused fatalities and extensive damage to Tarsin village and surrounding agricultural areas. The research addresses a critical knowledge gap created by more than two decades of armed conflict in Darfur, which has prevented on-the-ground investigations, by combining multisource geospatial data with deep convolutional neural networks (CNNs) to overcome accessibility constraints. The main findings are as follows:

- A landslide inventory of 350 events was developed from multi-temporal satellite imagery and combined with nine conditioning factors, including topography, hydrology, structural lineaments, vegetation cover, and anthropogenic influences. The CNN model, trained and validated using stratified k-fold cross-validation, demonstrated excellent predictive performance with an accuracy exceeding 0.97. Feature importance analysis highlighted elevation, curvature, and lineament density as the most influential factors, indicating the primary role of terrain morphology and structural weaknesses in slope failures.
- The resulting susceptibility map identifies high-risk areas concentrated in the central highlands and along drainage corridors (15–20% of the study area), medium-risk transitional zones (30–35%), and low-risk peripheral regions (50%). Predicted susceptibility shows strong spatial agreement with observed landslide occurrences, with Tarsin village located within high-hazard zones, underscoring the need for urgent risk management interventions.
- Key limitations include reliance on a remotely compiled landslide inventory without field verification, spatial resolution constraints of the conditioning factor datasets, and limited geological and soil data. Future studies should incorporate ground-based validation where security allows, higher-resolution remote sensing data, physically based stability modeling, temporal analysis of landslide occurrence, and expansion of conditioning factors to improve predictive reliability.
- This work demonstrates that effective landslide susceptibility assessment is feasible in conflict-affected regions by leveraging satellite data and machine learning, providing critical information for safeguarding communities where natural hazards intersect with socio-political vulnerabilities.

## Data Availability

The data that supports the findings of this study are available from the corresponding author upon reasonable request.
